# When Differential Descending Control of Speed Matters: Descending Modulation of A- versus C-Fiber Evoked Spinal Nociception

**DOI:** 10.3389/fpain.2022.910471

**Published:** 2022-06-09

**Authors:** Bridget M. Lumb, Lucy F. Donaldson

**Affiliations:** ^1^School of Physiology, Pharmacology and Neuroscience, University of Bristol, Bristol, United Kingdom; ^2^Pain Centre Versus Arthritis, University of Nottingham, Nottingham, United Kingdom

**Keywords:** descending pain modulatory systems, spinal nociception, A-nociceptor, C-nociceptor, PAG, hyperalgesia

## Abstract

Descending pain modulatory systems (DPMS) that originate within the brain and act to modulate spinal nociceptive transmission are a major determinant of the acute and chronic pain experience. Investigations of these systems in basic scientific research is critical to the development of therapeutic strategies for the relief of pain. Despite our best efforts, something is lost in translation. This article will explore whether this is due in part to a primary focus on sensory modality leading to a failure to differentiate between descending control of A- vs. C-fiber mediated spinal nociception.

## Introduction

Information about tissue damage is conveyed to the first synapse in pain pathways in the spinal cord by A-and C-fiber nociceptors. These nociceptors have different phenotypes [see (1) for review], convey different qualities of the pain signal (2–4), and have different roles in the development and maintenance of chronic pain (5–7). As such, they present different potential therapeutic targets. There is now good evidence that DPMS differentiate between information conveyed by Aδ- and C-fiber nociceptors. However, many *in vivo* studies (electrophysiological and behavioral) that are designed to test analgesic efficacy do not distinguish between A- and C-nociceptor evoked responsiveness. Indeed some may miss out any study of A-fiber nociceptors at all. This is a shortcoming and will affect interpretation and application of the findings. In this article, evidence will be presented that in acute pain DPMS primarily target responses to C-nociceptive inputs from the site of injury. However, in the transition to chronic (persistent) pain, there is a shift to descending pro-nociceptive control by DPMS of responsiveness to Aδ-nociceptive inputs from areas of secondary hyperalgesia (an area of hyperalgesia surrounding, or distant to, the site of injury) that results from central sensitization. This extends to descending control by clinically relevant prostanergic systems that originate in the midbrain periaqueductal gray and which are modulated by inhibition of cyclooxygenase (COX) enzymes by non-steroidal anti-inflammatory drugs (NSAIDS). The thesis presented here is that speed does indeed matter, because the differential descending control of A- vs. C-fiber evoked spinal nociception needs to be carefully considered in the design and interpretation of pre-clinical studies of pain mechanisms and when assessing the analgesic efficacy of potential therapeutic agents.

### Differential Descending Control of A- and C- Fiber Evoked Spinal Nociception: Role in Acute Pain

Initial studies by Waters and Lumb (8) identified differential descending control of spinal dorsal horn neurons with or without C-nociceptor inputs. Importantly, this and subsequent studies (9) demonstrated that, in naïve animals, the degree of inhibitory control from the periaqueductal gray (PAG) region of the midbrain is related to the magnitude of responses of individual dorsal horn wide dynamic range [WDR; otherwise known as Class 2 neurons (10)] to C-nociceptor activation. By contrast, neurons with weak, or an absence of, C-nociceptor inputs were “facilitated” by DPMS (8). Post-synaptic excitation of dorsal horn neurons from brainstem pain control centers may account for the facilitation (11, 12). However, analysis of the role of segmental mechanisms revealed that this “apparent” descending facilitation from the PAG was secondary to descending inhibition of those WDR neurons with C-fiber inputs and a resultant lifting of spinal segmental inhibition (8).

The different roles of A- and C-fiber nociceptors in both acute and chronic pain, together with the importance of their control by DPMS, led to the development of a non-invasive experimental approach first described by Yeomans et al. (13, 14). In their approach, either one or other of these populations of peripheral nociceptors can be preferentially activated using different rates of skin heating (15). A slow rate of skin heating was used to activate TRPV1 expressing, capsaicin sensitive, C-nociceptors and a fast rate to activate capsaicin insensitive A-fiber nociceptors [and see (16)]. This experimental tool has enabled more detailed studies of the underlying mechanisms, the behavioral significance, and the therapeutic potential of differential modulation of A- vs. C-fiber evoked spinal nociception by DPMS.

This approach in naïve animals confirmed differential control of A- vs. C-fiber evoked spinal nociception from the PAG, as assessed by descending influences on withdrawal reflexes (17) and on deep dorsal horn neurons (18). The differential control of deep dorsal horn neurons was not unique to the PAG but could be evoked from more rostral sites in the hypothalamus (19). Interestingly, studies that combined preferential activation of A- or C-nociceptors with the spinal induction of Fos protein indicated that differential descending control did not extend to the *superficial* dorsal horn, where the numbers of neurons activated by *both* A- or C-nociceptor stimulation were reduced by DPMS originating from the PAG (18). The significance of this latter finding remains to be established.

Importantly, preferential activation of A- vs. C-fiber nociceptors also revealed differences in the spinal processing of inputs from glabrous vs. hairy skin (20). Glabrous skin is innervated by a larger proportion of A-fiber nociceptors when compared to hairy skin (21) and a large proportion of C-polymodal nociceptors innervate the hairy skin of the rat hind foot (22, 23). This led to the hypothesis that during inflammation, the differences in primary afferent input from different skin types might differentially drive descending control systems and hence differentially affect the development of inflammatory hyperalgesia in the two skin types. This proved to be the case, with rapid inhibitory controls affecting C nociceptors in inflamed hairy, but not glabrous skin in rats (20). This observation is important given that hairy skin covers the vast majority of the body surface and the large number of studies that use reflex and neuronal responses to inputs from glabrous rather than hairy skin of the hindpaw to interrogate mechanisms of nociception and to assess efficacy of analgesic manipulations.

### Differential Descending Control of A- and C- Fiber Evoked Spinal Nociception: Role in Secondary Hyperalgesia

Preferential activation using different rates of skin heating has enabled mechanistic studies into the different roles of A- and C-fiber nociceptors in primary and secondary hyperalgesia. It is proposed that activity in C-nociceptors from the area of primary hyperalgesia at the site of injury triggers central sensitization leading to secondary hyperalgesia in surrounding tissue [e.g., (6)]. Observations in rodents (24, 25) confirmed reports from human studies by Treede and colleagues (6, 26) that it is indeed the facilitation of A-nociceptor-evoked responsiveness from an area distant to the site of injury that leads to secondary hyperalgesia. In other words, C-nociceptors innervating the area of primary hyperalgesia are the *facilitators* and A-nociceptors innervating the area of secondary hyperalgesia are the *facilitated* in the generation of secondary/distant hyperalgesia (6, 25, 26).

How does this relate to studies based on nociceptor modality? The most widely used methods for quantitative sensory testing of mechanical sensitivity in areas of primary and secondary hyperalgesia (typically von Frey hairs) activate both A- and C-fiber nociceptors, whereas the nature of most thermal stimuli (e.g., Hargreaves test) activate predominantly C-fiber nociceptors (26–30). It has been proposed that secondary hyperalgesia is evoked by mechanical and *not* by thermal stimuli (6, 7, 31–34) [but see (35)]. The use of the same modality of noxious stimulation (i.e., heat) to preferentially activate either A- or C-nociceptors from the same skin region clearly demonstrates thermal secondary hyperalgesia, but only when fast rates of skin heating are used to preferentially activate A-nociceptors (25). It would appear therefore that secondary hyperalgesia is not determined by the modality of noxious stimulation (thermal vs. mechanical) but rather by the type of nociceptor activated (A-fiber vs. C-fiber). Another factor with the capacity to confound the interpretation of findings is the method used to apply mechanical stimuli. For example, conflicting findings of the efficacy of the UVB model to generate secondary hyperalgesia in rodents were reported in studies that used either hand held (36) or automated von Frey devices (37). A direct comparison of the two methods suggests that the punctate stimulus produced by the hand held von Frey hairs activates A-fiber nociceptors to a greater extent than the blunt pressure produced by the automated device (36, 38, 39), which would account for the apparently conflicting findings.

### Differential Descending Control of A- and C- Fiber Evoked Spinal Nociception: Role in Centrally Acting Prostanoid-Targeted Analgesics

The PAG is a site of action of centrally acting analgesics, including opioids and COX-inhibitors such as non-steroidal anti-inflammatory drugs (NSAIDS). Interrogation of A- vs. C-fiber mediated spinal nociception has revealed important differences in their control by prostanoid mediated DPMS originating from the PAG. Crucial to the understanding of centrally mediated analgesia, the target of this control changes in the transition from acute to arthritic inflammatory pain. In acute pain, COX-1 sensitive systems target C-fiber-evoked spinal nociception. This control is inhibitory and is tonically active, indicating that endogenous, centrally acting, prostaglandins have a facilitatory action with the capacity to set the tone of acute pain (16). Prostaglandins exert their effects in the PAG through interactions with EP-receptors and, in an inflammatory model of arthritic pain, EP3 receptors mediate pro-nociceptive effects on A-fiber evoked spinal nociception from the area of secondary hyperalgesia. By contrast, C-fiber evoked responses from the same skin area remain unchanged (24). These data suggest that the analgesic actions of centrally penetrating NSAIDs in inflammation may be due, at least in part, to reduced descending facilitation of A-fiber mediated spinal nociception, rather than altered spinal processing, at which site EP receptors may also exert direct anti-nociceptive effects (40, 41).

## Discussion

If “speed” refers to A- vs. C-fiber evoked spinal nociception, the evidence presented here clearly indicates that speed does indeed matter in the context of descending control of spinal nociception and its implications for the transition to, and maintenance of, chronic pain. Rapidly conducting A-nociceptors and more slowly conducting C-nociceptors convey different qualities of the pain signal (first and second pain), have different phenotypes, play different roles in the initiation and maintenance of chronic pain and differentially innervate glabrous vs. hairy skin. Evidence supports the view that activated C-nociceptors act as facilitators that trigger central changes including spinal facilitation of A-nociceptive inputs from areas adjacent, and distant, to the site of injury, leading to secondary hyperalgesia. The role of alterations in DPMS in the transition from acute to chronic pain is well-established [e.g., (42, 43)]. The studies presented here highlight the importance of understanding the contributions of differential control of A- and C-nociceptor evoked spinal nociception by DPMS in this transition. To improve translational value and the development of therapeutic strategies the interpretation of previously generated data and the design of future experimental studies need to differentiate between contributions of DPMS to A-fiber mediated vs. C-fiber mediated processes, and not concentrate solely on nociceptive modality.

## Author Contributions

BL and LD: conceptualization, funding acquisition, and writing, review and editing. Both authors contributed to the article and approved the submitted version.

## Funding

Work from the laboratories of BL and LD at University of Bristol (UK) was supported by the Medical Research Council (UK), the Biotechnology & Biological Sciences Research Council (UK), the Wellcome Trust, University of Bristol (UK), MRC, and BBSRC Case studentships supported by GlaxoSmithKline.

## Conflict of Interest

The authors declare that the research was conducted in the absence of any commercial or financial relationships that could be construed as a potential conflict of interest.

## Publisher's Note

All claims expressed in this article are solely those of the authors and do not necessarily represent those of their affiliated organizations, or those of the publisher, the editors and the reviewers. Any product that may be evaluated in this article, or claim that may be made by its manufacturer, is not guaranteed or endorsed by the publisher.
